# Astilbin Protects Against Ischemic Stroke by Regulating ERK1/2/CREB/p90RSK Signaling and Ferroptosis-Related SLC7A11/ACSL4/GPX4 Axis: Insights from Network Pharmacology, Multi-Omics, and Molecular Dynamics

**DOI:** 10.3390/ijms27114749

**Published:** 2026-05-25

**Authors:** Chang Jin, Yue Zhang, Bing Li, Zhifeng Cheng, Meizhu Zheng, Weihua Dong, Kai Song, Yongxing Ai

**Affiliations:** 1 College of Life Science, Changchun Normal University, Changchun 130032, China; j1504408478@163.com (C.J.); 15942907284@163.com (Y.Z.); 18544359016@163.com (B.L.); czf20170726@163.com (Z.C.); songkai@ccsfu.edu.cn (K.S.); 2 The Central Laboratory, Changchun Normal University, Changchun 130032, China; 3 School of Geographical Sciences, Changchun Normal University, Changchun 130032, China; 4 College of Animal Science, Jilin University, Changchun 130062, China; aiyx@jlu.edu.cn

**Keywords:** ischemic stroke, Astilbin, network pharmacology, multi-omics, mechanism of action

## Abstract

Ischemic stroke is an acute cerebrovascular disease with high disability and morbidity. However, therapeutic approaches are restricted by a narrow time window for reperfusion. Astilbin has various pharmacological activities and good therapeutic potential against ischemic stroke and neurodegenerative diseases. Nevertheless, Astilbin’s mechanism of action remains unclear. Here, we used an integrated strategy that includes network pharmacology, omics validation, and functional verification. Potential targets of Astilbin were predicted using SwissTargetPrediction and PharmMapper, and cross-analyzed with IS-related genes from multiple databases. GO/KEGG enrichment analyses showed that Astilbin synergistically regulates stroke-associated pathways (e.g., MAPK, AGE-RAGE). Combined transcriptomic and metabolomic assays confirmed that Astilbin ameliorates OGD/R-induced oxidative stress and metabolic disorders by modulating the MAPK and ferroptosis pathways. Molecular docking and dynamics simulations revealed that Astilbin has high affinity for core targets (ERK1/2, CREB, p90RSK, MMP9) and binds stably to MMP9. Using an OGD/R-injured neuronal-like PC12 cell line, in vitro assays confirmed that Astilbin alleviates oxidative stress, calcium overload, lipid peroxidation, and intracellular iron levels, while also modulating apoptosis- and inflammation-related genes. Overall, this study has established a comprehensive pharmacological framework for the use of Astilbin against IS, clarified its multi-target, multi-pathway neuroprotective mechanisms of action, and provided evidence for its potential in the treatment of IS.

## 1. Introduction

As an acute cerebrovascular disease, ischemic stroke (IS), more commonly known as cerebral infarction, occurs when a sudden interruption of cerebral blood supply causes ischemic damage to focal brain areas, ultimately leading to neurological deficits [1]. It is defined as a cerebrovascular disorder that causes neurological impairment due to ischemia and hypoxia in the brain, triggered by various etiologies. IS involves a complex pathophysiological process, and its major pathogenic mechanisms include inflammatory response [2], cell apoptosis or necrosis [3], excitotoxicity [4], autophagy [5], oxidative stress [6], as well as ferroptosis and cuproptosis [7,8,9]. At present, the primary goal of acute-phase treatment is to achieve early cerebral reperfusion, primarily via intravenous thrombolysis (tPA) and mechanical thrombectomy. However, these two most effective therapies are subject to strict time constraints, and the majority of patients miss the optimal treatment window due to delayed medical attention [1]. Therefore, overcoming the time-window limitation, alleviating post-reperfusion injury, and promoting long-term neurological recovery have become key scientific issues in ischemia research.

Identification of lead compounds for stroke treatment from natural medicines represents a crucial strategy for research and development. Active components of traditional Chinese medicines (TCMs) exhibit multi-targeted properties and lower side effects, demonstrating significant potential for anti-inflammatory, antioxidant, and neuroprotective effects that may address the limitations of current therapeutic approaches.

Astilbin, a flavonoid compound, is mostly found in Chinese herbal plants, such as Smilax glabra, Engelhardtia roxburghiana, and Hydrangea paniculata. Previous studies have documented that Astilbin displays multiple bioactivities, encompassing anti-inflammatory, antioxidative, immune-regulating, and anti-apoptotic properties [10], suggesting that it has good therapeutic potential against diseases, such as IS and neurodegenerative diseases [6]. Astilbin mitigates cerebral ischemia–reperfusion injury through multiple mechanisms: it dampens neuroinflammation by blocking the TLR4/MyD88/NFκB axis, modulates calcium homeostasis to limit excitotoxicity, stimulates the Nrf2 pathway to counteract oxidative stress, and regulates apoptotic cascades to reduce neuronal death. Moreover, astilbin improves cognitive performance in Alzheimer’s disease models and attenuates CD4^+^ T cell-driven inflammation [6,11,12,13]. Whether Astilbin has a clear therapeutic effect against IS and its specific neuroprotective mechanism of action remains unclear, thus requiring further research. The present study delivers a fundamental scientific underpinning and novel molecular blueprints to support the creation of new anti-stroke pharmaceuticals.

To solve this problem, our study adopts an approach that combines computational prediction, omics verification, and functional confirmation. Initially, we used databases, such as SwissTargetPrediction, PharmMapper, and GeneCards, for target prediction. After merging and removing duplicates, we obtained the potential action targets of Astilbin. We retrieved gene information from GeneCards, OMIM, PharmGkb, and DisGeNET, intersected the results, and identified key targets that Astilbin may regulate. Functional enrichment analysis showed that Astilbin synergistically regulates multiple stroke-related pathways, such as MAPK (Mitogen-Activated Protein Kinase), IL-17 (Interleukin-17), AGE-RAGE (Advanced Glycation End Products–Receptor for Advanced Glycation End Products), and atherosclerosis, targeting core genes such as MMP9, TGFB1, and IGF1R. Combined with transcriptomic and metabolomic verification, we found that Astilbin significantly improved oxidative stress and energy metabolism disorders induced by OGD/R (Oxygen and Glucose Deprivation/Reperfusion) -mediated ischemic injury by regulating the MAPK signaling (ERK1/2 (extracellular regulated protein kinases 1/2)-CREB (cAMP response element-binding protein)-p90RSK (p90 ribosomal S6 kinase) axis) and ferroptosis-related (SLC7A11–ACSL4–GPX4 axis) pathways. In addition, key target complexes were verified by using molecular docking and molecular dynamics simulation. Dynamics simulation of the MMP9 complex showed good binding stability and structural compatibility, indicating that these targets exert neuroprotective effects through multi-target synergistic action.

**Figure 1:**
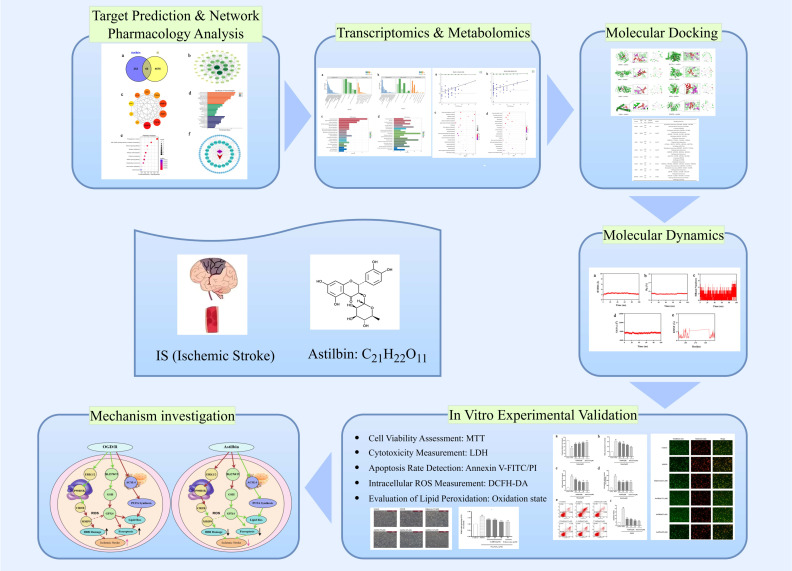
Flowchart depicting Astilbin-mediated neuroprotection in a model of IS. Arrows show the stepwise progression of the research workflow.

Finally, the OGD/R-induced injury model of PC12 cells was used to assess functional activities of LDH concentration, Ca^2+^ concentration, ROS concentration, apoptosis rate, mitochondrial membrane potential, lipid peroxidation level, and iron ion levels. To further elucidate the potential mechanisms of Astilbin against IS, we examined the mRNA and protein levels of key pathway components using quantitative real-time PCR (RT–qPCR) and Western blotting. Overall, our findings present a multi-level mechanistic basis for Astilbin’s protective effects against IS, thereby affording crucial insight into its translational feasibility as a multi-target neuroprotective compound (Figure 1).

## 2. Results

### 2.1. Network Pharmacological Analysis of Astilbin Against IS

**Figure 2:**
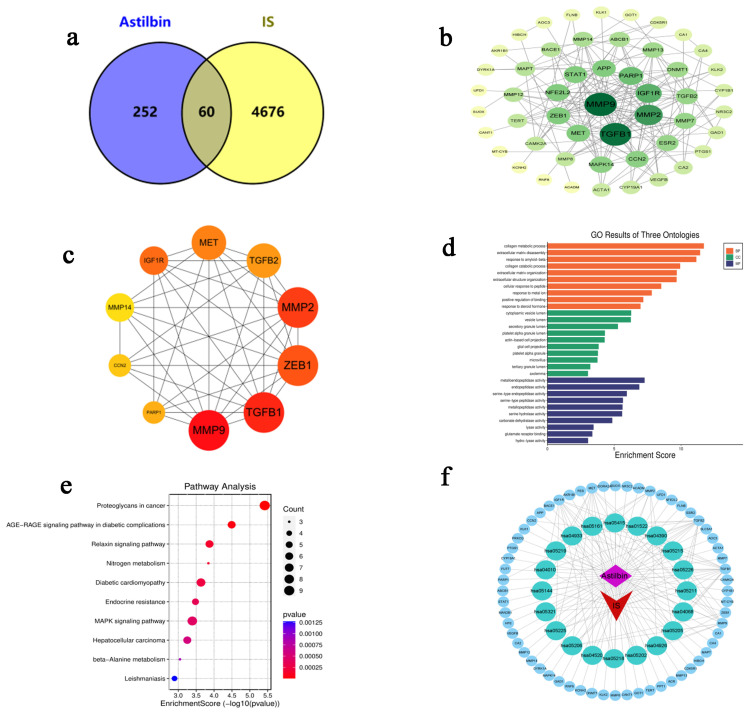
Target and enrichment landscape of Astilbin against ischemic stroke (IS). (**a**) Overlap between drug-relevant and disease-relevant targets displayed in a Venn diagram (60 shared targets). (**b**) PPI network of the 60 intersecting targets. (**c**) Hub gene ranking based on MCC algorithm (top 10 shown). (**d**) GO analysis: top 10 biological processes (orange), cellular components (green), and molecular functions (blue). (**e**) Enriched KEGG pathways (top 20 listed). (**f**) Gene–pathway association network.

Target prediction was performed using databases SwissTargetPrediction, PharmMapper, and GeneCards. Gene information related to IS was retrieved from PharmGkb, OMIM, DisGeNET, and GeneCards databases, leading to the identification of 60 overlapping genes (Figure 2a). A PPI (Protein–Protein Interaction) network with 60 nodes and 177 edges was obtained using String12.0 (Figure 2b). The top 10 hub genes (MMP9, TGFB1, MMP2, IGF1R, PARP1, APP, STAT1, NFE2L2, MET, and ZEB1) were identified using the MCC (Maximal Clique Centrality) algorithm in the cytoHubba plugin (Figure 2c). The targets identified by GO enrichment analysis were largely enriched in biological processes involving collagen metabolism, organization of the extracellular matrix, and its disassembly (Figure 2d). KEGG pathway enrichment analysis indicated significant enrichment of numerous pathways, including proteoglycans in cancer, diabetic cardiomyopathy, the MAPK signaling pathway, AGE-RAGE signaling in diabetic complications, fluid shear stress and atherosclerosis, lipid and atherosclerosis, IL-17 signaling, TNF signaling, and necroptosis (Figure 2e).

Construction of a pathway-gene interaction network showed that Astilbin connects to multiple core target gene nodes by acting on multiple key signaling pathways that are closely related to inflammatory responses, immune regulation, cell apoptosis, and vascular remodeling. Among the outer targets, several of them (APP, IGF1R, BACE1, MMP2, MAPK14, STAT1, VEGFB, MMP9) are in relative hub positions, suggesting that they may be the key molecular basis for Astilbin’s involvement in the occurrence and development of IS (Figure 2f). Overall, the results indicate that Astilbin does not act through a single target but rather exerts its effects by synergistically modulating multiple stroke-related signaling pathways and their core genes. Consequently, it produces a comprehensive intervention effect on IS by influencing interrelated processes, including the inflammatory cascade, neuronal damage, and vascular dysfunction. These findings provide a basis for elucidating Astilbin’s potential mechanism in the prevention and treatment of IS.

### 2.2. Multi-Omics Analysis of Astilbin Against IS

#### 2.2.1. Transcriptomic Analysis of Astilbin Against IS

**Figure 3:**
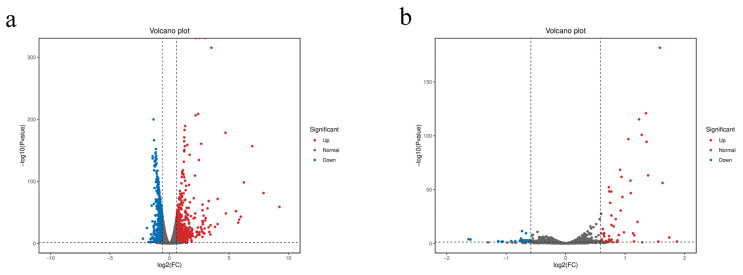

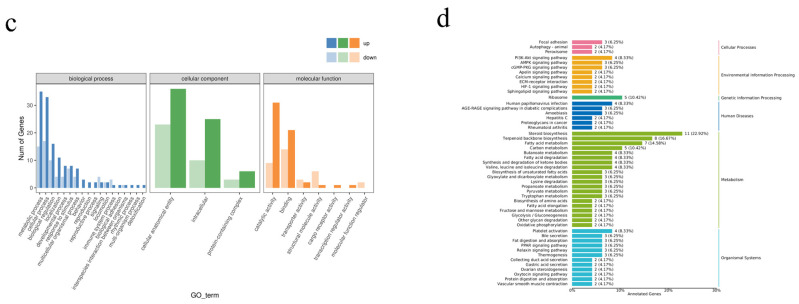
Transcriptome-based elucidation of Astilbin’s protective mechanisms in IS. (**a**) Volcano plot of DEGs (control vs. model). (**b**) Volcano plot of DEGs (model vs. Astilbin). (**c**) Leading 10 GO annotations from DEGs (model vs. Astilbin). (**d**) KEGG pathway terms from DEGs (model vs. Astilbin).

To further explore the potential effects of Astilbin on IS, a transcriptomic analysis of PC12 cells was conducted. As shown in Figure 3a, a comparison between the model and control groups revealed 106 differentially expressed genes (DEGs), of which 53 were upregulated and 53 downregulated. In the Astilbin group relative to the model group, a total of 1928 DEGs were identified, including 854 upregulated and 1074 downregulated genes (Figure 3b). To further explore the mechanism of Astilbin in IS, GO and KEGG enrichment analyses were subsequently performed. Astilbin, as revealed by GO enrichment analysis, primarily affected biological processes that were tightly connected to reproduction, reproductive process, growth, positive regulation of cell death, and other related terms (Figure 3c). As previously noted, cell death in PC12 cells is closely associated with IS. Accordingly, the results suggest that Astilbin may ameliorate IS by modulating PC12 cell death, which aligns with earlier findings. KEGG pathway enrichment analysis revealed that the anti-IS targets of Astilbin were significantly clustered in the PI3K/Akt signaling pathway, the MAPK cascade, and several additional pathways (Figure 3d). Integrative analysis further indicates that Astilbin’s pharmacological effect on IS is mechanistically linked to the regulation of the PI3K/Akt cascade.

#### 2.2.2. Metabolomic Analysis of Astilbin Against IS

**Figure 4:**
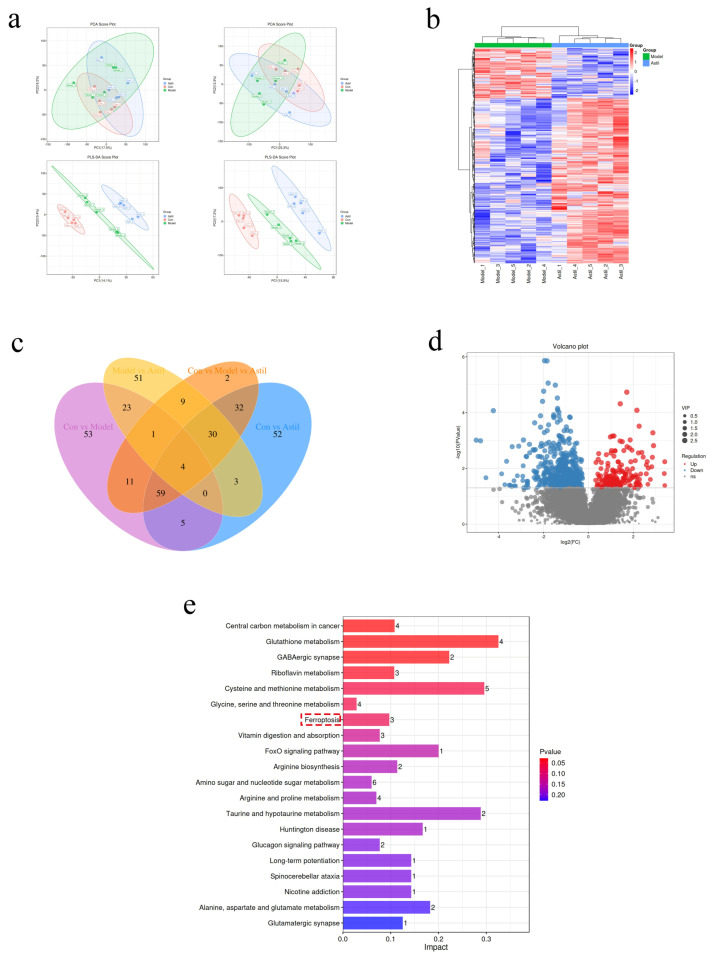
Differential metabolite profiling across the control, model, and Astilbin groups. (**a**) PLS−DA and OPLS-DA score plots acquired in positive/negative ion modes. (**b**) Heatmap representation of clustered metabolic features. (**c**) Venn diagram showing overlaps of group-specific differential metabolites. (**d**) Volcano plot: Astilbin vs. model group. (**e**) KEGG enrichment: control vs. model. The red dashed box highlights the ferroptosis pathway for emphasis.

PLS-DA (partial least squares discriminant analysis) and OPLS-DA (orthogonal partial least squares discriminant analysis) analyses demonstrated clear separation of metabolic profiles among the control, model (OGD/R model), and Astilbin-treated (OGD/R + Astilbin) groups, suggesting that Astilbin effectively reversed metabolic dysregulation induced by OGD/R simulated ischemic injury. (Figure 4a). A total of 6611 differentially expressed metabolites were identified. Cluster analysis combined with differential metabolite screening (VIP > 1, *p* < 0.05, PFDR < 0.05) yielded 663 significantly dysregulated metabolites in the model relative to the control group. Of these, 489 exhibited upregulation, and 144 exhibited downregulation.

The Astilbin group displayed 458 metabolites with altered expression levels when set against the model group; 162 of these were increased, whereas 296 were decreased. A Venn diagram illustrates the distribution of these differential metabolites across the experimental groups (Figure 4b–d). KEGG pathway enrichment analysis showed marked enrichment of the ferroptosis pathway (Figure 4e), emphasizing its critical contribution to OGD/R-induced cellular injury. Notably, this pathway was not significantly enriched when comparing the model group with the Astilbin-treated group, suggesting that Astilbin effectively modulates ferroptosis-related metabolic networks.

### 2.3. Integrative Network Pharmacology, Transcriptomics, and Metabolomics Analysis

We conducted multi-omics integration analysis using the Biomarker Cloud Platform and the R language (v4.2.1). We co-mapped differentially expressed genes from the transcriptome and differential metabolites from the metabolome to KEGG pathways, identified common pathways by pathway co-mapping and highly correlated gene-metabolite networks (|r| > 0.8), and then cross-validated core targets predicted by network pharmacology with differentially expressed genes from the transcriptome. Finally, we integrated predicted and experimentally enriched pathways, focusing on key signaling networks.

**Figure 5:**
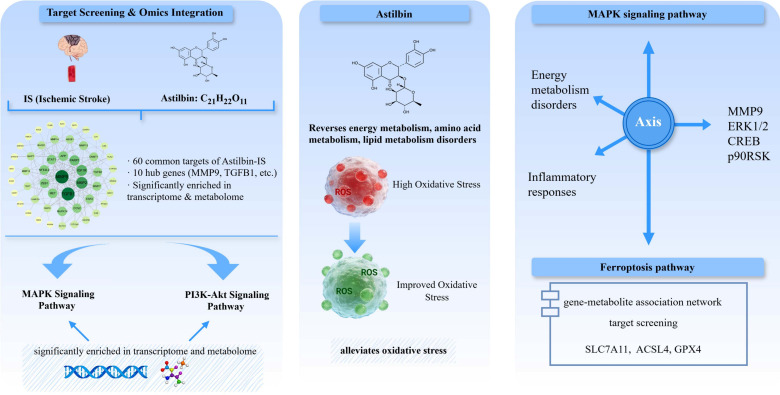
Multi-omics integration and Astilbin mechanism analysis in OGD/R model injury.

Multi-omics integration validated 60 common targets screened by network pharmacology and key hub genes, such as MMP9, TGFB1, MMP2, IGF1R, PARP1, APP, STAT1, NFE2L2, MET, and ZEB1. At the level of signaling routes, MAPK and PI3K-Akt, predicted by network pharmacology, were consistently supported by KEGG enrichment analyses of transcriptomics and metabolomics, suggesting that these pathways play important roles in the process of Astilbin intervening in OGD/R-induced cell injury. Astilbin can significantly reverse disorders in energy, amino acid, and lipid metabolisms caused by OGD/R injury, and alleviate oxidative stress. Our results from a multi-omics joint analysis showed that in the OGD/R model, the MAPK signaling pathway plays a central regulatory role and is identified as the core regulatory axis, closely related to metabolic disorders and inflammatory responses, as illustrated in Figure 5. Meanwhile, the ferroptosis pathway was important in the gene-metabolite association network and target screening, suggesting that Astilbin may function synergistically to regulate the MAPK (MMP9, ERK1/2, CREB, p90RSK) and the ferroptosis (SLC7A11, ACSL4, GPX4) pathways.

Overall, network pharmacology combined with multi-omics integration analysis clarified the logical path for mechanistic verification. Astilbin may regulate the MAPK signaling pathway (MMP9, ERK1/2, CREB, p90RSK) and synergistically inhibit the ferroptosis pathway (SLC7A11, ACSL4, GPX4), thereby improving metabolic disorders, oxidative stress, and inflammatory damage induced by the OGD/R model in multiple aspects. This, in turn, exerts a neuroprotective effect.

### 2.4. Molecular Docking of Astilbin with Therapeutic Targets

**Figure 6:**
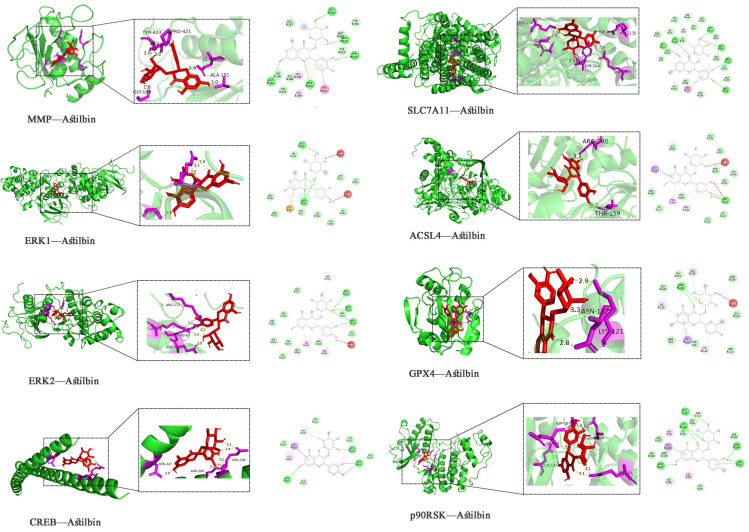
Astilbin docking with key targets. Binding modes and interactions of Astilbin with MMP9, ERK1, ERK2, CREB, SLC7A11, ACSL4, GPX4, and p90RSK, highlighting binding conformations and key residues.

Following a literature review, molecular docking was performed between Astilbin and its core targets, including ERK1, ERK2, CREB, p90RSK, MMP9, SLC7A11, ACSL4, and GPX4. The docking analysis (summarized in Table 1) and 3D binding diagrams (Figure 6) demonstrated that all binding energies were below −7 kcal/mol. This suggests a good theoretical interaction between Astilbin and the selected targets. Among them, ERK1, ERK2, SLC7A11, and MMP9 exhibited stronger binding affinities with Astilbin, suggesting they may be key candidate mediators of its neuroprotective function. Notably, Astilbin showed the strongest binding energies with ERK1 (−10.5 kcal/mol) and ERK2 (−9.5 kcal/mol), implying a prominent putative binding interaction at the structural level with these two core kinases in the MAPK signaling pathway.

On one hand, it may potentially regulate ferroptosis by binding to ACSL4, a positive regulator of this process. On the other hand, its binding to SLC7A11 (a cystine transporter) and GPX4 (a core antioxidant enzyme) may help maintain their biological activities. Furthermore, studies have shown that active MMP9 interacts with GPX4 and glutathione reductase, leading to reduced GPX4 expression and activity, thereby identifying stromal MMP9 as a key regulator of ferroptosis. In summary, by integrating network pharmacology, multi-omics analysis, and molecular docking, we propose a tentative mechanistic hypothesis: the neuroprotective effects of Astilbin may be mediated by regulation of the MAPK-ERK signaling pathway and the SLC7A11/ACSL4/GPX4 ferroptosis pathway. Accordingly, subsequent cell and molecular biology experiments were performed to further validate the above mechanistic prediction.

### 2.5. Md Simulations of the Therapeutic Target Complex of Astilbin

#### 2.5.1. Basic Conformational Stability Analysis of Molecular Dynamics

**Figure 7:**
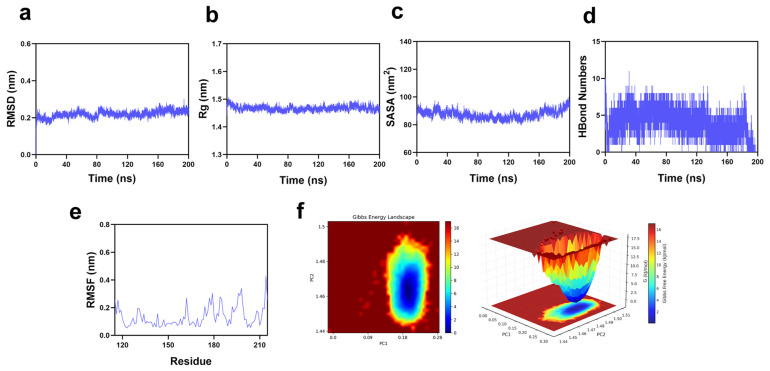
Molecular dynamics simulation analysis of the MMP9–Astilbin complex. (**a**) Time evolution of the root-mean-square deviation (RMSD) for the complex. (**b**) Variation in the radius of gyration (Rg) throughout the simulation. (**c**) Temporal profile of the solvent-accessible surface area (SASA). (**d**) Number of intermolecular hydrogen bonds formed during the trajectory. (**e**) Per-residue root-mean-square fluctuation (RMSF) of the complex. (**f**) Free energy landscape (FEL) constructed from the MD ensemble.

To provide further support for the molecular docking outcomes and to evaluate how stably the ligand binds to the receptor, we performed molecular dynamics simulations on the MMP9–Astilbin complex system of the MMP9 target gene, which shows good binding free energy and is implicated in network pharmacology, transcriptomics, and metabolomics. The structural stability of the protein–ligand complex throughout the molecular dynamics trajectory was assessed by monitoring the root-mean-square deviation (RMSD). The RMSD trajectory indicated that the complex system reached equilibrium by 80 ns, after which it fluctuated stably around a mean value of 0.24 nm (Figure 7a). Thus, this small molecule exhibited high binding stability with the target protein. The radius of gyration (Rg) of the complex varied slightly during the entire simulation, suggesting that the complex maintained a relatively compact structure without obvious expansion or contraction (Figure 7b). In addition, the solvent-accessible surface area (SASA, solvent accessible surface area) indicated that the accessible area on the protein surface did not change significantly upon ligand binding, indicating that ligand binding did not significantly alter the overall three-dimensional structure of the protein (Figure 7c). Hydrogen bond analysis demonstrated that the ligand–target protein interaction involved a variable number of hydrogen bonds (0–11) during the simulation, and the complex predominantly featured approximately six bonds. This result demonstrates that the ligand establishes favorable, stable hydrogen-bond interactions with the target protein (Figure 7d). According to the root-mean-square fluctuation (RMSF)—an indicator of protein residue flexibility—the complex exhibited relatively low fluctuation values (predominantly below 0.35 nm), suggesting low flexibility and high stability (Figure 7e). The Free Energy Landscape (FEL) is shown in Figure 7f, which presents the free-energy surface results for the complex. The figure employs a color gradient to encode free energy, with the x-axis denoting PC1 (root-mean-square deviation, RMSD) and the y-axis representing PC2 (radius of gyration, Rg), both expressed in nanometers. In the free energy landscape, red regions correspond to high-energy states, whereas blue regions denote low-energy basins. Notably, a pronounced energy minimum was observed at approximately PC1 = 0.25 and PC2 = 1.485, suggesting that the associated conformations are thermodynamically more stable.

#### 2.5.2. Binding Free Energy and Key Residue Analysis

**Figure 8:**
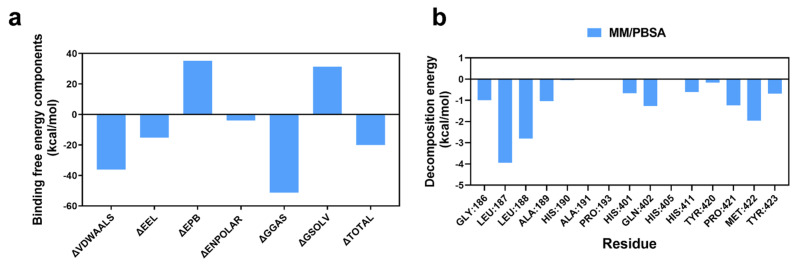
Binding free energy and key residue contribution analysis of the MMP9−Astilbin complex. (**a**) Binding free energy, calculated by the MM/PBSA method. (**b**) Amino acid residue contributions of the complex.

The binding free energy of the small molecule to the target protein was subsequently estimated from the complex conformation using the MM/PBSA approach (Figure 8a). The binding free energy of the complex was determined to be −19.99 kcal/mol. The negative sign denotes that the molecule binds to the target protein, and a greater negative value implies tighter binding. Subsequently, the amino acid residues that contribute critically to small-molecule binding in the complex were identified and analyzed. Figure 8b highlights the key residues providing high contributions to small-molecule binding: LEU187, LEU188, MET422, GLN402, PRO421, ALA189, GLY186, HIS401, TYR423, and HIS411. This indicates that these amino acid residues may play an important role in the catalytic process.

Based on the above results, the small molecule exhibited a stable binding conformation and good structural compatibility with MMP9 at the in silico level. The formed hydrogen-bond network supports favorable ligand–protein interaction characteristics, but cannot confirm that Astilbin acts as a direct functional inhibitor of MMP9. Further enzyme activity and biochemical assays are still required to verify its regulatory effect on MMP9 activity.

### 2.6. Neuroprotective Effect of Astilbin in the OGD/R Model

#### 2.6.1. Morphological Changes of PC12 Cells

**Figure 9:**
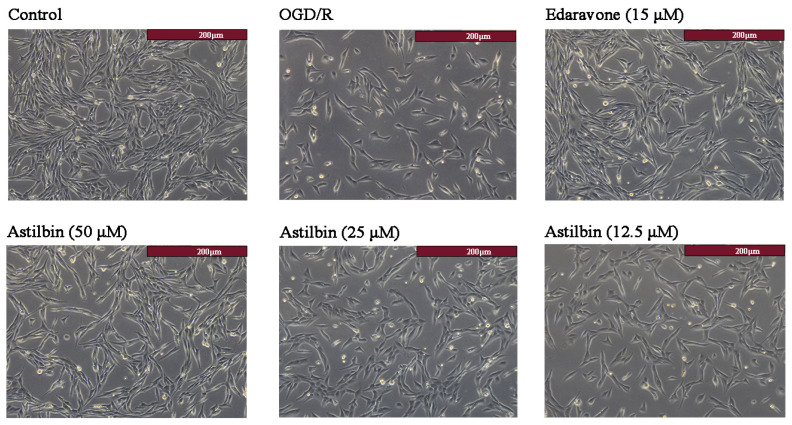
Morphology of PC12 cells in each group (×200).

Visualization via bright-field microscopy uncovered that PC12 cells in the blank control group had normal morphology, clear synapses, and were interconnected to form a network structure. In the model group relative to controls, cells displayed shrinkage, a decrease in abundance, and marked destruction of synaptic connections. Cell damage in the Edaravone group and the Astilbin drug protection group was improved compared with the model group—i.e., the number of cells increased—and the synaptic structures recovered. A concentration-dependent cytoprotective effect of Astilbin was observed. Treatment with 50 μM restored near-typical cell morphology, elevated cell density, preserved structural integrity, and enhanced attachment (Figure 9).

#### 2.6.2. Comprehensive Protective Effect of Astilbin in the OGD/R Model

**Figure 10:**
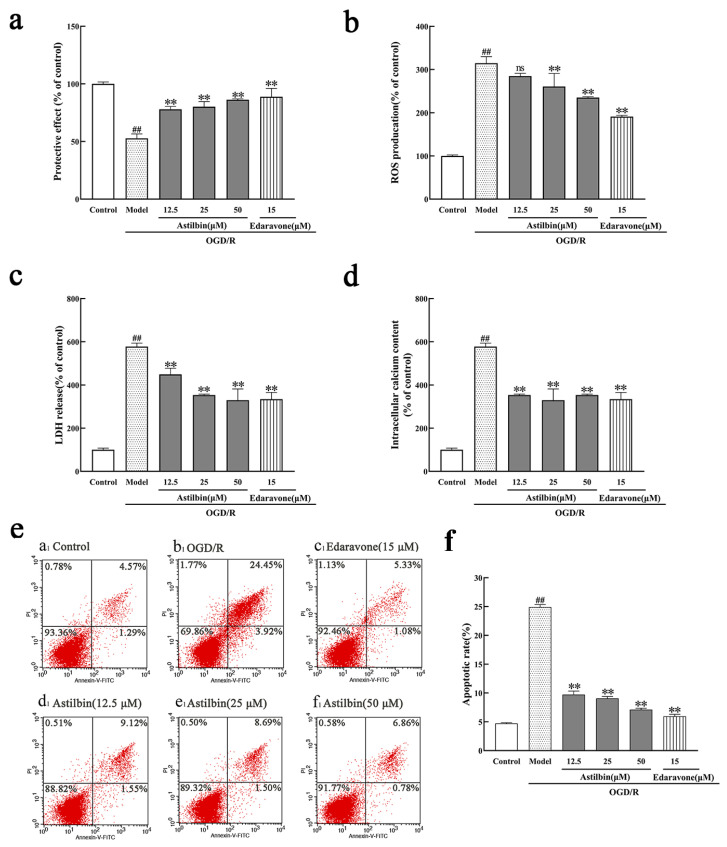
Astilbin exerts protective and anti-apoptotic actions against OGD/R injury. (**a**) Cell survival rates. (**b**) ROS content. (**c**) LDH release. (**d**) Ca^2+^ levels. (**e**) Flow cytometry plots of apoptosis in each group (**f**) Histogram of apoptosis rate for each group. ## *p* < 0.01, compared to control, ** *p* < 0.01 compared with the OGD/R group.

As shown in Figure 10a, PC12 cells in the model group had a significantly reduced survival rate (*p* < 0.01), whereas Astilbin treatment markedly improved cell survival (*p* < 0.01). Additionally, Figure 10b–d revealed that the OGD/R treatment group exhibited substantially elevated levels of ROS, LDH, and Ca^2+^ (*p* < 0.01), all of which were notably decreased by Astilbin at each dose (12.5, 25, 50 μM, *p* < 0.01, respectively). Similar protective effects were observed with the positive control, Edaravone. In addition, flow cytometric assessment (Figure 10e) substantiated that OGD/R caused extensive apoptosis, as shown by a notable elevation in the percentage of apoptotic cells (Figure 10f). The percentage of apoptotic cells decreased dose-dependently with Astilbin: 9.12% at 12.5 μM, 8.69% at 25 μM, and 6.86% at 50 μM, while the positive control (Edaravone) achieved an apoptosis rate of 5.33%. All Astilbin concentrations, along with the positive control, dramatically decreased apoptosis rates relative to the model group (*p* < 0.01). Accordingly, Astilbin potently inhibits OGD/R-mediated apoptosis in PC12 cells and confers neuroprotective effects (Figure 10).

#### 2.6.3. Astilbin Can Reduce the Ferrous Ion Concentration in PC12 Cells Induced by OGD/R

**Figure 11:**
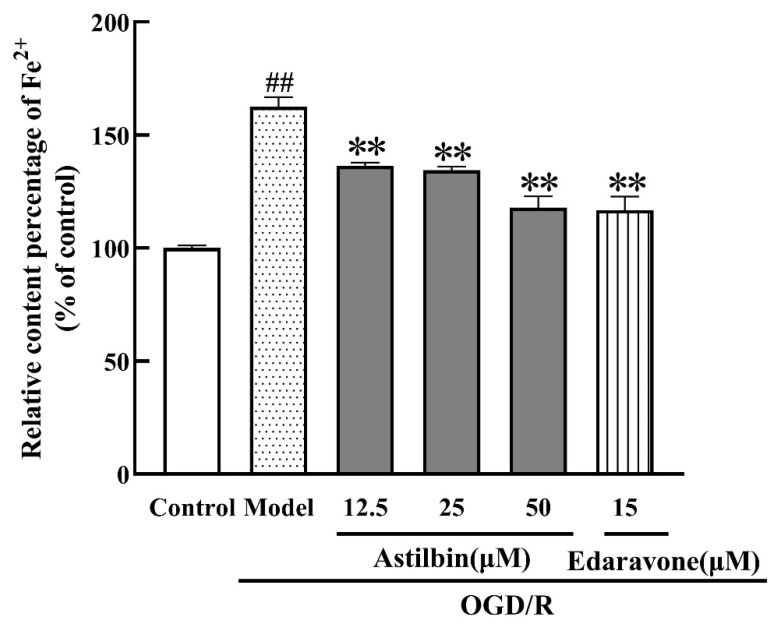
Influence of Astilbin on the relative level of Fe^2+^ in PC12 cells subjected to OGD/R. Data are presented as mean ± SD (n = 3). ## *p* < 0.01 versus the blank control group; ** *p* < 0.01 versus the OGD/R model group.

Ferrous ion (Fe^2+^) detection revealed that OGD/R significantly elevated the relative intracellular Fe^2+^ content in PC12 cells compared with the untreated control (## *p* < 0.01), demonstrating that OGD/R can cause ferrous iron accumulation. Compared with the model group, Astilbin treatment markedly reduced the relative intracellular Fe^2+^ level (** *p* < 0.01), suggesting that Astilbin effectively suppressed the OGD/R-induced elevation of ferrous iron and attenuated its aberrant accumulation. These results suggest that Astilbin has an improving effect on the iron homeostasis imbalance in PC12 cells caused by OGD/R (n = 3) (Figure 11).

#### 2.6.4. Astilbin Reduces OGD/R-Induced Lipid Peroxidation in PC12 Cells

**Figure 12:**
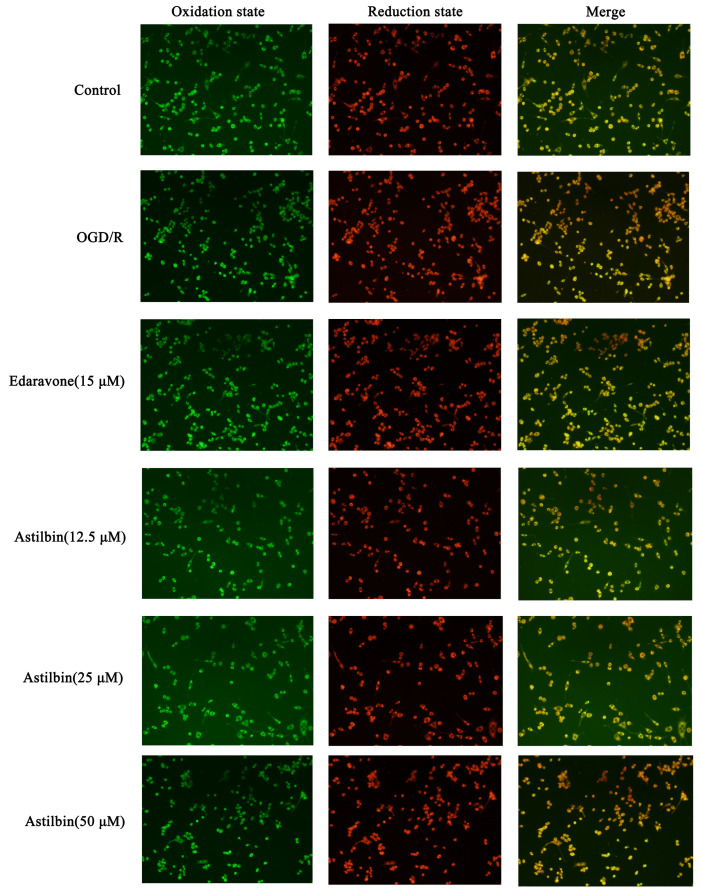
Influence of Astilbin on lipid peroxidation in OGD/R-challenged PC12 cells.

Upon OGD/R-induced ischemic injury model establishment, the level of lipid peroxidation in PC12 cells increased significantly. Probing lipid peroxidation with a ratiometric fluorescent sensor showed that the OGD/R group had a significantly higher green/red fluorescence ratio than the control, indicating a shift toward the oxidized state and a loss of the reduced form. The merged image was overall more yellow-green, suggesting that OGD/R can induce increased oxidation of cell membrane lipids, showing obvious lipid peroxidation damage, which is a typical phenotypic change related to ferroptosis (Figure 12).

Edaravone (15 μM) administration improved the OGD/R-triggered lipid peroxidation. Compared to the OGD/R group, the Edaravone group showed attenuated green fluorescence, enhanced red fluorescence, and a merged image with diminished yellow-green intensity, approximating the control. This indicates that Edaravone can inhibit OGD/R-induced lipid peroxidation and exert an antioxidant protective effect.

Treatment with Astilbin also alleviated lipid peroxidation damage caused by OGD/R, and showed an apparent dose-dependent improvement. Compared with the OGD/R group, the Astilbin 12.5 μM group had a decreased signal of the oxidized state (green) and an enhanced signal of the reduced state (red). When the dose was increased to 25 μM and 50 μM, the green signal was further weakened, the red signal was further enhanced, and the merged image was overall closer to that of the control group. These results indicate that Astilbin markedly attenuates OGD/R-induced lipid peroxidation, thereby mitigating oxidative stress injury in cells.

### 2.7. Experimental Validation of Predicted Target Proteins

#### 2.7.1. Western Blotting Verifified ERK1/ERK2/CREB/p90RSK and Ferroptosis Signaling Pathways

**Figure 13:**
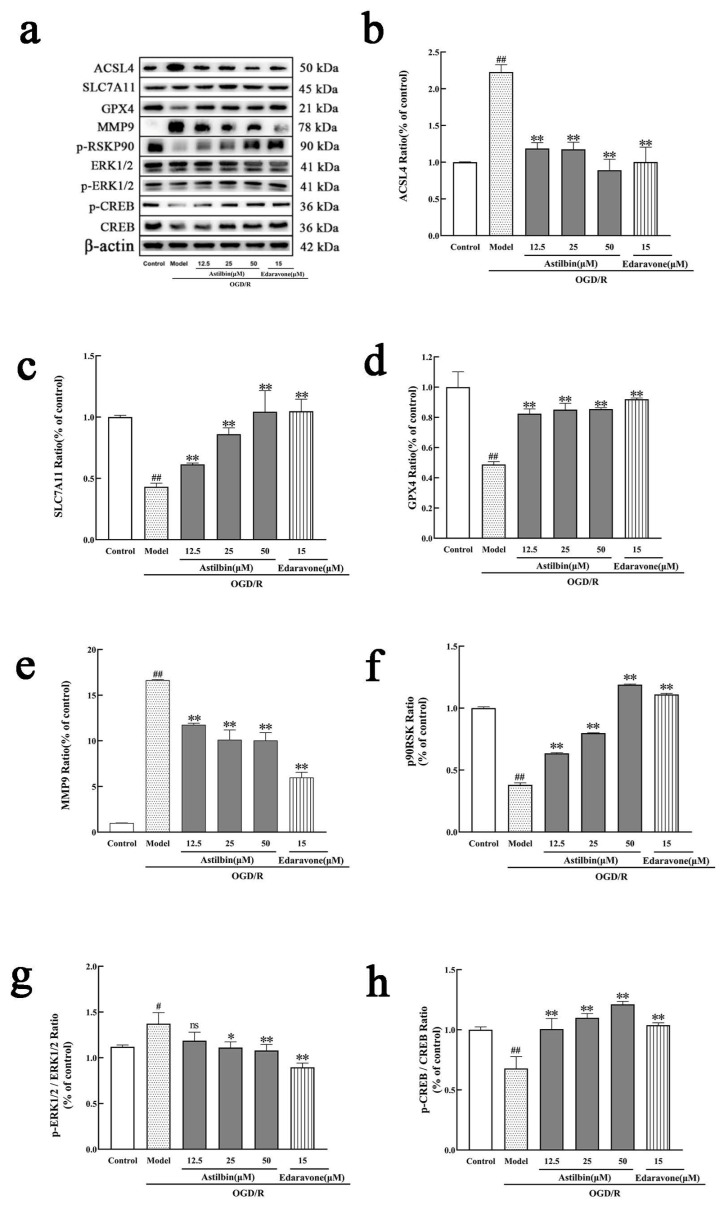
Influence of Astilbin on the protein expression of ACSL4, SLC7A11, GPX4, MMP9, p90RSK, ERK1/2, p-ERK1/2, CREB, and p-CREB in OGD/R-induced PC12 cells. (**a**) Representative Western blot images of target proteins. (**b**) ACSL4 protein levels. (**c**) SLC7A11 protein levels. (**d**) GPX4 protein levels. (**e**) MMP9 protein levels. (**f**) p90RSK protein levels. (**g**) p-ERK1/2/ERK1/2 ratio. (**h**) p-CREB/CREB ratio. ## *p* < 0.01 versus the blank control group; n.s. if # *p* > 0.05; * *p* < 0.01, ** *p* < 0.01 versus the model group.

Protein expression analysis by Western blot indicated that, in the OGD/R group, ERK1, ERK2, p90RSK, CREB, and ACSL4 were significantly lower than in the blank control, while MMP9, SLC7A11, and GPX4 were significantly higher. These results are consistent with our transcriptome data and also support the hypothesis that Astilbin exerts neuroprotective effects by regulating key proteins. The neuroprotective action of Astilbin is achieved through coordinated regulation of the ERK1/ERK2, CREB, p90RSK signaling networks and ferroptosis-linked proteins MMP9, SLC7A11, ACSL4, and GPX4, thus delineating one of its central molecular mechanisms (Figure 13).

#### 2.7.2. Real-Time Fluorescent Quantitative PCR of ERK1/ERK2/CREB/p90RSK and Ferroptosis Signaling Pathways

**Figure 14:**
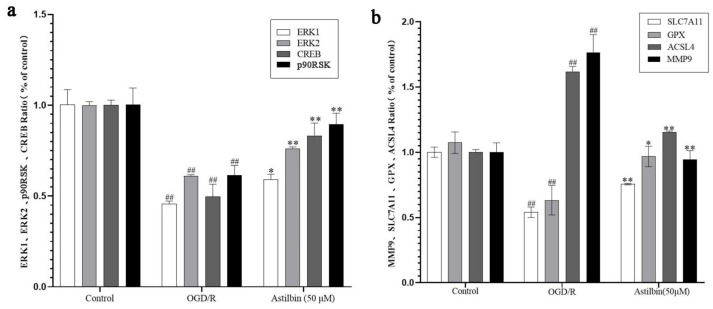
Relative mRNA expression levels of ERK1, ERK2, CREB, p90RSK, SLC7A11, ACSL4, GPX4, MMP9. (**a**) Relative mRNA levels of ERK1, ERK2, CREB and p90RSK; (**b**) Relative mRNA expressions of SLC7A11, ACSL4, GPX4 and MMP9. Data are represented as mean ± SD (n = 3). * *p* < 0.05, ** *p* < 0.01 versus the blank control group; ## *p* < 0.01 versus the OGD/R model group.

We performed RT-qPCR to detect mRNA expression of genes related to these pathways to verify that Astilbin exerts its anti-IS effect by regulating signaling pathways, such as ERK1/ERK2/CREB/p90RSK/MMP9 and SLC7A11/ACSL4/GPX4. The mRNA levels of ERK1, ERK2, p90RSK, CREB, and ACSL4 were significantly lower in the OGD/R group than in the blank control, while those of MMP9, SLC7A11, and GPX4 were significantly higher. Astilbin treatment significantly upregulated the expression of ERK1, ERK2, p90RSK, CREB, and ACSL4 compared with the model group, while downregulating MMP9, SLC7A11, and GPX4 (Table 2 and Table 3). This indicates that Astilbin regulates transcription levels of key genes in the ERK, CREB, and ferroptosis-related signaling pathways, which is likely an important molecular mechanism of action by which Astilbin exerts its neuroprotective effect (Figure 14a,b).

## 3. Discussion

The present study focused on the neuroprotective effect of Astilbin, a natural flavonoid compound, against IS. By integrating network pharmacology prediction, multi-omics analysis, molecular docking, and molecular dynamics simulation, together with in vitro cell experiments, we systematically explored the multi-level mechanism underlying Astilbin protection against IS.

The pathological progression of IS is complicated and involves inflammatory response, oxidative stress, and multiple biological processes. Compared with single-target drugs, natural flavonoids represented by Astilbin exhibit superior advantages via multi-target synergistic regulation [14,15]. Network pharmacology predicted 60 intersection targets between Astilbin and IS, and PPI network analysis further screened key hub genes such as MMP9, TGFB1, IGF1R, PARP1, STAT1, and NFE2L2. Enrichment analysis showed a strong association of the target set with inflammatory pathways and matrix remodeling, findings that are concordant with the pathological characteristics of IS.

Transcriptomic combined with metabolomic results revealed that Astilbin exerted extensive regulatory effects on OGD/R-injured PC12 cells. This intervention markedly regulated energy, amino acid, and lipid metabolism, attenuated oxidative stress, recovered mitochondrial function and energy homeostasis, and suppressed excessive ROS production, ultimately ameliorating the metabolic derangements triggered by ischemic injury [16,17,18,19].

The core mechanisms underlying Astilbin’s neuroprotection in IS, as indicated by network pharmacology and multi-omics analyses, encompass activation of the MAPK–ERK–CREB/p90RSK signaling [20,21], inhibition of dysregulated MMP9 [22], and modulation of the ferroptosis-related SLC7A11/ACSL4/GPX4 axis to reduce oxidative stress [23,24,25]. These signaling pathways interact and coordinate with each other to jointly mediate neuroprotection, among which MMP9 acts as a critical linker connecting MAPK signaling and ferroptosis progression. Astilbin attenuated inflammation and oxidative stress while preserving intracellular and extracellular homeostasis, processes intimately linked to the regulation of ferroptosis.

Molecular docking showed that Astilbin exhibited high binding affinity to core targets, including MMP9, ERK1/2, CREB, and GPX4, with all binding energies below −7 kcal/mol. Molecular dynamics simulation confirmed that the Astilbin–MMP9 complex maintained a stable conformational behavior over 50 ns, as reflected by steady RMSD, Rg, and SASA fluctuations, and persistent hydrogen bond formation. These computational results supported the stable binding and multi-target characteristics of Astilbin at the in silico level [26,27,28]. Nevertheless, molecular docking only provided preliminary interaction clues and cannot confirm direct activation or inhibition of target proteins; further biochemical and enzyme activity verification is still required.

In vitro experiments showed that Astilbin had no obvious cytotoxicity to PC12 cells below 50 μM. It markedly elevated cell viability, reduced LDH release, relieved intracellular Ca^2+^ overload, and inhibited ROS accumulation and neuronal apoptosis [29,30]. qRT-PCR and Western blotting further validated that Astilbin significantly upregulated ERK1/2, CREB, and p90RSK expression to facilitate neuronal repair against ischemia–reperfusion injury [31]. Meanwhile, Astilbin downregulated ACSL4, upregulated SLC7A11 and GPX4, suggesting its protective role in ferroptosis via maintaining glutathione metabolism [25,32].

As the top hub gene in the PPI network, MMP9 is closely correlated with neuronal injury, blood–brain barrier disruption, cerebral edema, and reperfusion damage, and is therefore a key therapeutic target for IS [33]. Our results confirmed that Astilbin effectively inhibited MMP9 expression, and molecular docking plus dynamics simulation further verified its stable binding with MMP9, providing a structural basis for suppressing MMP9 activity.

Mechanistically, the MAPK pathway occupies a central position in Astilbin-mediated neuroprotection and has been widely recognized as a promising therapeutic target for IS [34]. As a key downstream effector of MAPK–ERK signaling, CREB participates in neuronal survival and neural functional recovery after ischemia [35]. The upregulation of CREB by Astilbin was accompanied by reduced apoptosis and improved mitochondrial function, enabling neurons to tolerate ischemic injury via activating the MAPK–ERK–CREB/p90RSK axis.

Ferroptosis is a newly defined regulated cell death widely involved in neurodegeneration and ischemic injury [36]. SLC7A11/GPX4 axis is essential for maintaining glutathione homeostasis and lipid peroxide clearance, while abnormal ACSL4 expression promotes ferroptotic progression [37,38,39]. These results demonstrated that Astilbin regulated the SLC7A11/ACSL4/GPX4 pathway, attenuated lipid peroxidation, and reinforced the antioxidant defense to inhibit ferroptosis.

Importantly, these pathways are not independent of each other. Active MMP9 was reported to interact with GPX4 and regulate ferroptosis [40], while MAPK activation acts as a common upstream signal modulating autophagy and ferroptosis [41]. Such crosstalk further explained the multi-pathway synergistic characteristics of Astilbin.

**Figure 15:**
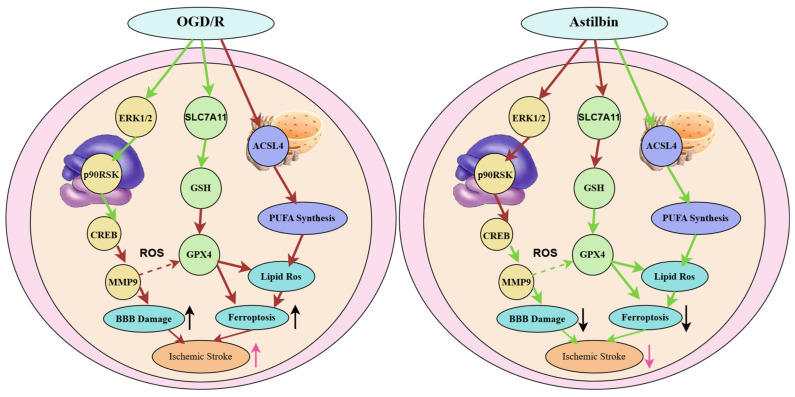
Schematic illustration of the mode of action of Astilbin against ischemic stroke. Red arrows indicate promotion; green arrows indicate inhibition; upward arrows indicate promotion, and downward arrows indicate inhibition.

In summary, this study demonstrated that Astilbin exerted neuroprotective effects against IS via multi-target and multi-pathway regulation: activating the MAPK–ERK–CREB pro-survival cascade, restraining MMP9-related inflammation and blood–brain barrier damage, and regulating ferroptosis metabolic networks. This integrated in vitro and in silico study provides preliminary experimental and theoretical evidence for the neuroprotective mechanism of Astilbin under oxidative stress [42] (Figure 15).

## 4. Materials and Methods

### 4.1. Target Identification and Network Pharmacology Analysis

A network pharmacology strategy was adopted to elucidate the neuroprotective mechanisms of Astilbin in ischemic stroke (IS). Astilbin’s chemical structure was downloaded from PubChem [43] and fed into SwissTargetPrediction, PharmMapper, and GeneCards for target fishing. Duplicates were removed during data integration, yielding the final set of predicted targets [44,45]. With “IS” as the search term, disease-associated genes were collected from the GeneCards, OMIM, PharmGKB, and DisGeNET databases [45,46,47,48]. UniProt was employed to standardize and deduplicate the target list. Overlapping targets were then identified via Venny2.1 [49] and imported into STRING (with a high-confidence cutoff of 0.7) for PPI network construction [50]. Hub genes were screened using Cytoscape (v3.8.0) and its cytoHubba plugin [51]. Functional annotation analysis for GO terms and KEGG pathways was performed using DAVID (*p* ≤ 0.01), followed by visualization through Cytoscape 3.10.1 and integrated web-based platforms [52].

### 4.2. Based on Multi-Omics Analysis

#### 4.2.1. Collection and Analysis of Transcriptome Samples

Total RNA was extracted from high-quality samples with cell count ≥1 × 10^6^ and intact morphology. RNA purity, concentration, and integrity were assessed with a NanoDrop 2000 spectrophotometer and an Agilent 2100 Bioanalyzer. mRNA was enriched by Oligo(dT) magnetic beads and randomly fragmented, followed by cDNA synthesis, end repair, A-tailing, adapter ligation, and size selection to construct sequencing libraries, which were then PCR amplified [53,54,55]. PE150 sequencing was performed on the Illumina NovaSeq 6000 platform [55]. After filtering, 110.94 Gb of high-quality clean data were generated, with at least 5.56 Gb per sample and Q30 ≥ 95.92%. Clean reads were mapped to the rat reference genome (Rattus_norvegicus.Rnor_6.0) using HISAT2 with a mapping rate of 95.73–96.65%. Gene expression was quantified by StringTie in FPKM units. Differentially expressed genes (DEGs) were identified using edgeR with thresholds of |fold change| ≥ 1.5 and FDR < 0.05. Data normalization and batch correction were applied to minimize systematic variations. Principal component analysis (PCA) and inter-sample correlation matrices demonstrated strong reproducibility across biological replicates, with pairwise Pearson correlation coefficients (r^2^) exceeding 0.8. DEGs in multiple comparison groups (such as C_vs_M, M_vs_A) were identified and classified into upregulated and down-regulated categories [56,57]. The DEGs were subjected to GO and KEGG enrichment to determine their biological functions. Enrichment analysis was conducted with ClusterProfiler (v4.0.5) based on hypergeometric tests, with significance defined as *p*.adjust < 0.05 to identify significantly enriched biological processes, molecular functions, cellular components, and metabolic pathways [58,59]. A total of 15 samples were included, comprising three groups (C, M, A) with five biological replicates each.

#### 4.2.2. Collection and Analysis of Metabolomics Samples

Cell metabolites were extracted using pre-chilled extraction buffer, and supernatants were collected after grinding, ultrasonication, and centrifugation. Non-targeted metabolic profiling was accomplished using ultra-performance liquid chromatography–quadrupole time-of-flight mass spectrometry (UPLC-QTOF-MS) in positive and negative electrospray ionization modes. Progenesis QI was employed for raw data processing, encompassing peak detection, alignment, and total peak area normalization. We identified metabolites by comparing their *m*/*z* values, retention times, and MS/MS spectra with entries in the METLIN, HMDB, and LipidMaps databases, as well as an in-house library of authentic standards. Differential metabolites were screened using combined criteria of VIP > 1 (OPLS-DA) and *p* < 0.05 (Student’s *t*-test). KEGG pathway enrichment analysis was performed using ClusterProfiler (v4.0.5) with *p*.adjust < 0.05 as a significance threshold, and visualized by bubble plots and network diagrams [60]. Quality control assessments, including stable base peak chromatograms, tight clustering of QC samples in PCA, and CV < 15% in QC samples, guaranteed the reliability of the metabolomic data.

### 4.3. Transcriptome-Metabolome Integrated Pathway and Network Analysis

To integrate transcriptomic and metabolomic data, DEGs and differential metabolites (DMs) were jointly projected onto KEGG pathway maps. Common regulatory pathways were identified by pathway co-mapping and gene-metabolite association networks (Spearman correlation analysis, |r| > 0.8) [61]. Core targets predicted by network pharmacology were cross-validated with DEGs from the transcriptome, and the pathways enriched by prediction and experiments were integrated to focus on key signaling networks [15]. The analysis was mainly completed on the Biomarker Cloud Platform and in the R language environment, using toolkits such as clusterProfiler, Cytoscape 3.10.1, and MetaboAnalystR [62].

### 4.4. Molecular Docking Analysis

To investigate interactions between Astilbin and target proteins, we used molecular docking in silico simulations. Astilbin’s CAS identifier was retrieved via the TCMSP platform, while the corresponding three-dimensional conformation was fetched from the PubChem compound database. The target protein structures (Homo sapiens) were obtained from the PDB, preprocessed in PyMOL 2.5.0 to eliminate non-protein atoms, and then subjected to docking on the CB-Dock2 server (5 independent runs). From the AutoDock Vina 1.2.0 results, the top-ranked conformation (lowest binding free energy) was adopted as the final binding mode. All structural images were prepared using PyMOL 2.5.0 [63].

### 4.5. Molecular Dynamics Simulations

Molecular dynamics simulations were conducted in GROMACS 2022.2 [64]. Force field parameters were generated using the GROMACS pdb2gmx utility and the AutoFF web server [65]. The receptor was described by the Amber99SB-ILDN force field [66], while the ligand was parameterized with GAFF2 [67]. The ligand topology was built using Sobtop 1.0 (dev3.1), and restrained electrostatic potential (RESP) charges were assigned. We solvated the system in a TIP3P water box and subsequently introduced Na^+^ and Cl^−^ ions (0.15 M) via the gmx genion tool to ensure electroneutrality. The PME method was used for long-range electrostatic interactions, and the LINCS algorithm was applied for bond constraints. We carried out energy minimization by applying 3000 steepest descent steps and then 2000 conjugate gradient steps. Energy minimization was carried out in three sequential stages: initially, the solute was restrained while the solvent was minimized; next, both the solute and counterions were fixed during a second minimization step; and lastly, an unrestrained minimization of the whole system was performed. Production MD was then conducted in the NPT ensemble (310 K, 1 bar) for 100 ns with a 2 fs time step. Temperature and pressure were regulated by a Nosé–Hoover thermostat (τ = 0.1 ps) and a Parrinello–Rahman barostat (τ = 2.0 ps), respectively. Post-simulation analysis was carried out with GROMACS utilities: RMSD (g_rmsd), RMSF (g_rmsf), hydrogen bonds (g_hbond), radius of gyration (g_gyrate), solvent-accessible surface area (g_sasa), and free energy landscape (g_sham) were computed according to established protocols [68].

### 4.6. In Vitro Experiments

#### 4.6.1. Reagents and Antibodies

Astilbin (purity 99.76%) and the positive control edaravone (EDA) were purchased from MedChemExpress (Monmouth Junction, NJ, USA). High-glucose DMEM for cell culture and fetal bovine serum (FBS) were purchased from Sangon Biotech (Shanghai, China). Penicillin-streptomycin mixture (double antibody) was purchased from Hyclone, UT, USA. Other agents utilized to measure LDH detection kit, ROS detection kit, Ca^2+^ fluorescent probe kit, cell apoptosis detection kit, BCA kit, Western blotting reagents, lipid peroxidation detection kit, iron ion and ferrous ion detection kit, and cell lysis buffer obtained from Beyotime (Shanghai, China). Sodium dithionite, MTT, and a protein size marker (10–180 kDa) were provided by Solarbio Science and Technology (Beijing, China). EBSS was from Feijing, Fuzhou, China. The primary antibodies used in Western blotting include β-actin, ERK1/2, p-ERK1/2, p90RSK, p-p90RSK, CREB, p-CREB, MMP9, SLC7A11, ACSL4, and GPX4, all from Abcam (Cambridge, UK). SLC7A11, ACSL4, and GPX4 were from Abclonal (Wuhan, Hubei, China). The secondary antibody (HRP goat anti-rabbit IgG) was from Dingguo Changsheng, Beijing, China. Millipore Corporation, USA, served as the supplier for the Immobilon-P roll PVDF membrane (Billerica, MA, USA). RNA extraction kit, genome-removing cDNA first-strand synthesis premix, and fluorescent quantitative PCR premix reagents were purchased from Tiangen, Beijing, China. Acrylamide (30%) and 1X PBS were from Lanjieke Company, Beijing, China.

#### 4.6.2. In Vitro Evaluation of the Neuroprotective Effect of Astilbin

PC12 cells (sourced from Shanghai, China) were grown in complete medium consisting of 10% FBS and 1% double antibiotics at 37 °C under 5% CO_2_. The medium was replaced regularly according to growth status, and cells were digested and passaged when they adhered to the wall and reached 80% confluency [69]. Then, an in vitro ischemic injury model was established. We used sodium dithionite as the modeling agent and adopted a chemical induction method to establish the OGD/R model. The original medium was discarded and replaced with a hypoxia-inducing solution containing 10 μmol/L sodium dithionite for 2 h to simulate ischemic injury. After that, the hypoxia-inducing solution was poured out, and normal medium was used for further culturing for 24 h to simulate reperfusion injury [70]. The experimental groups consisted of: (i) control (no treatment), (ii) OGD/R alone, (iii) OGD/R + Astilbin at 12.5, 25, or 50 μM, and (iv) OGD/R + Edaravone (15 μM) as a positive control. All treatments were initiated 1 h prior to OGD/R and continued during reoxygenation.

#### 4.6.3. Morphological Observation

Following treatment, PC12 cells were stored at 37 °C for 24 h. An inverted microscope was used to evaluate morphological changes in the designated groups (n = 6).

#### 4.6.4. Determination of Cell Viability by MTT Assay

Exponentially growing PC12 cells were seeded into 96-well plates. After adherence, they were assigned to the following treatment groups: Group I—the AST concentration gradient group; the culture media contained different concentrations of AST (1.5625, 3.125, 6.25, 12.5, 25, 50, 100, 200 μM). Group II—positive control; the culture media contained 15 μM edaravone. Group III—blank control; the culture media contained no drugs. Each group had six replicate wells. Following 24 h of drug exposure, the MTT assay was used to evaluate cell viability. All groups were re-perfused for 24 h; then 10 μL of MTT (5 g/L) was added to each well, followed by incubation at 37 °C for 4 h. After discarding the supernatant, the formazan crystals were dissolved in 100 μL DMSO. Absorbance at 490 nm was then measured with a spectrophotometer, and relative cell viability was calculated [69]. The experiment was independently repeated three times.

#### 4.6.5. LDH Release Assay

LDH release was quantified with a commercial assay kit (Beyotime) following 24-h reoxygenation, as per the provided protocol. The procedure was performed as per the manufacturer’s instructions. Briefly, after treatment, the plate was mixed gently, spun at 250 g for 5 min, and the medium was collected and added to the matching wells of a new 96-well plate. Following the addition of the LDH reaction mixture and incubation at room temperature for 30 min, absorbance was determined at 450 nm with a microplate reader.

#### 4.6.6. Detection of Ca^2+^ Concentration

After 24 h of reoxygenation, the intracellular Ca^2+^ content in PC12 cells was detected according to the instructions provided in the Beyotime Fura-3/AM kit.

#### 4.6.7. Detection of Reactive Oxygen Species (ROS) Levels

After 24 h of reoxygenation, ROS levels were determined using the Beyotime ROS assay kit according to the manufacturer’s instructions. After aspirating the culture medium, cells were incubated with 10 µM DCFH-DA working solution at 37 °C for 40 min in the dark. Afterwards, cells were washed three times with phosphate-buffered saline (PBS) to remove excess probes. Fluorescence was recorded with a multi-functional microplate reader (excitation 488 nm, emission 525 nm), and ROS levels were presented as relative fluorescence units (RFU).

#### 4.6.8. Detection of Iron Ions in PC12 Cells

PC12 cells were reoxygenated for 24 h, after which ferric and ferrous ions were detected as directed by the kit manufacturer.

#### 4.6.9. Detection of Lipid Peroxidation in PC12 Cells

After 24 h of reoxygenation, lipid peroxidation was assessed in PC12 cells using a commercial assay kit according to the manufacturer’s instructions.

#### 4.6.10. Detection of Apoptosis Rate

After 24 h of reoxygenation, cell apoptosis was detected according to instructions provided in the cell apoptosis detection kit.

#### 4.6.11. Western Blotting

Following the addition of lysis buffer (200 µL), cells were left on ice for 20 min. Cells were then gently mixed by pipette, transferred to a centrifuge tube, and centrifuged at 4 °C and 13,000× *g* for 5 min. The supernatant was collected as the protein sample for later use.

After electrophoresis and transferance, the membrane was placed in primary antibody solution (1:1000 dilution) and incubated overnight at 4 °C for β-actin, ERK1/2, p-ERK1/2, p90RSK, p-p90RSK, CREB, p-CREB, MMP9, SLC7A11, ACSL4, and GPX4. After washing, blots were incubated with peroxidase-conjugated secondary antibodies for 45 min. Proteins were detected by enhanced chemiluminescence (ECL). Quantification of band intensity was carried out using QuantityOne (v4.6.8, Bio-Rad, Hercules, CA, USA), and all values were normalized against β-actin.

#### 4.6.12. Real-Time Fluorescence Quantitative PCR

Total cellular RNA was extracted according to instructions provided in the RNA extraction kit (Tiangen, Beijing, China), and cDNA was obtained by reverse transcription. The amplification reaction was performed on ice. In the experiment, GAPDH was used as the internal reference gene. The relative expression of the target gene mRNA was calculated by comparing the Ct values of the target gene and GAPDH based on the 2^−ΔΔCt^ method.

#### 4.6.13. Methods of Data Statistics and Analysis

Statistical analyses were performed using GraphPad Prism 9. Results are expressed as the mean ± standard deviation (SD) of at least three independent biological replicates. Normal distribution and homogeneity of variance were routinely assessed prior to statistical comparison. Multiple group comparisons were performed using one-way ANOVA followed by Tukey’s post hoc test. We adjusted for multiple testing in the transcriptomic and metabolomic datasets to limit the false discovery rate (FDR). A *p*-value below 0.05 was considered indicative of a statistically significant difference.

## Figures and Tables

**Table 1:** Astilbin docking with key target proteins: protein data, binding affinity, RMSD, and interaction types (hydrophobic, H-bond, salt bridge, π-stacking).

Protein	PDB ID	Ligands	Affinity (kcal/mol)	RMSD	Bonding Interaction
MMP9	1GKC	Astilbin	−9.2	0.609 Å	Hydrophobic Interactions (LEU188B, HIS190B, VAL398B) Hydrogen Bonds Interactions (GLN402B) π-Stacking (HIS401B)
ERK1	2ZOQ	Astilbin	−10.5	0.605 Å	Hydrophobic Interactions (ARG96B, GLU98B) Hydrogen Bonds Interactions (ARG96B, VAL100B, GLY102B, ARG104B) Salt Beidges (ARG96B, HIS195A)
ERK2	1PME	Astilbin	−9.5	0.613 Å	Hydrophobic Interactions (THR185A, TYR187A, VAL188A, LYS203A) Hydrogen Bonds Interactions (GLN66A, ARG67A, ARG70A, ARG148A, ARG172A) Salt Beidges (ARG172A)
CREB	2LXT	Astilbin	−7.2	0.607 Å	Hydrophobic Interactions (ARG240B, ASN247B, LYS299A) Hydrogen Bonds Interactions (SER239B, ASN247B, ARG296A, ARG300A)
SLC7A11	7CCS	Astilbin	−9.3	0.610 Å	Hydrogen Bonds Interactions (ARG135B, LYS198B, SER330B, ARG396B) π-Stacking (TYR251B) Salt Beidges (LYS198B)
ACSL4	6OZ1	Astilbin	−8.3	0.614 Å	Hydrophobic Interactions (THR183A, PRO320A, PRO345A, LEU346A, GLU681A, PHE689A) Hydrogen Bonds Interactions (ARG380A, GLU681A)
GPX4	2GS3	Astilbin	−7.0	0.608 Å	Hydrophobic Interactions (ARG9B, ILE122B, ILE129B) Hydrogen Bonds Interactions (ARG9B, LYS121B, GLY128B, ASN132B)
p90RSK	2WNT	Astilbin	−8.8	0.608 Å	Hydrophobic Interactions (VAL39D, ALA52D, MET108D, LEU156D) Hydrogen Bonds Interactions (LYS114D) Salt Beidges (LYS114D)

**Table 2:** Relative mRNA expressions of ERK1, ERK2, p90RSK, and CREB.

Group	ERK1	ERK2	p90RSK	CREB
Control	1.002 ± 0.048	1 ± 0.011	1.003 ± 0.053	1 ± 0.016
OGD/R model	0.457 ± 0.008 ^##^	0.610 ± 0.004 ^##^	0.614 ± 0.031 ^##^	0.496 ± 0.040 ^##^
Astilbin (50 μM)	0.612 ± 0.033 *	0.761 ± 0.005 **	0.896 ± 0.034 **	0.831 ± 0.040 **

VS. the blank control group, * *p* < 0.05, ** *p* < 0.01; VS. the model group, ^##^ *p* < 0.01 (n = 3).

**Table 3:** Relative mRNA expression statistics of SLC7A11, ACSL4, GPX4, and MMP9.

Group	SLC7A11	ACSL4	GPX4	MMP9
Control	1.001 ± 0.022	1.000 ± 0.012	1.074 ± 0.047	1.002 ± 0.040
OGD/R model	0.540 ± 0.023 ^##^	1.617 ± 0.023 ^##^	0.633 ± 0.065 ^##^	1.765 ± 0.079 ^##^
Astilbin (50 μM)	0.756 ± 0.003 **	1.154 ± 0.005 **	0.968 ± 0.045 *	0.943 ± 0.040 **

VS. the blank control group, * *p* < 0.05, ** *p* < 0.01; VS. the model group, ^##^ *p* < 0.01 (n = 3).

## Data Availability

The datasets and original data supporting the findings of this study are available within the article. Additional information related to this work may be obtained from the corresponding authors upon reasonable request.
